# Social and Political Dimensions of Disseminating Research Findings on Emerging Zoonotic Viruses: Our Experience in Sierra Leone

**DOI:** 10.9745/GHSP-D-20-00321

**Published:** 2021-09-30

**Authors:** Dorothy Peprah, James Bangura, Mohamed Vandi, Harold Thomas, Monica Dea, Anton Schneider, Kendra Chittenden

**Affiliations:** a U.S. Agency for International Development, Washington, DC, USA.; b University of California Davis, PREDICT Program, Freetown, Sierra Leone.; c Ministry of Health and Sanitation, Freetown, Sierra Leone.; d United States Agency for International Development, Freetown, Sierra Leone.

## Abstract

Disseminating research findings on emerging zoonotic viruses is a complex and sensitive process, particularly in contexts with histories of outbreaks. It requires an operational framework that considers the social and political context of stakeholders aiming to empower people to protect their health, while also supporting government leaders to advance global health security.

## Key Messages

- We discuss the complexities and challenges in disseminating research findings on the discovery of a new ebolavirus in bats in Sierra Leone, particularly in light of the previous outbreaks.
- This experience pointed to the tensions that can arise between discoveries of emerging viruses with zoonotic potential and their impact on the people in direct proximity to the ecosystems in which they are found as well as on the governments charged with protecting the health of those people.
- Future research may benefit from considering the following: (1) prioritize dissemination of findings as a distinct step within a project with a detailed plan, (2) understand and support the role of government leaders in the dissemination process, and (3) prioritize the perspectives and potential reactions of communities when framing information and recommendations.

## INTRODUCTION

Sharing research results with communities is a key stage of implementing global health research. This stage is widely accepted not only as essential to conducting ethical, fair research but also as integral to improving the relevance and impact of research for participants and their communities.^1^^,^^2^ The various activities that compose results sharing may be conducted as part of a research project’s ongoing community engagement strategy or dissemination plan. However, although sharing research results with communities is widely regarded as a best practice in global health research, little systematic guidance exists for considering the social and political implications of those findings alongside government leaders and as part of community-level dissemination strategy. Although the discipline is growing and an increasing number of scholars are interested in dissemination and implementation science, the focus is on clinical research and provider uptake.^3^ Little consideration has been given to how to manage the dissemination of research findings that may be perceived as negative and may require negotiation not only at the community level but also at the subnational and national levels.

This issue is especially true of research projects on emerging zoonotic diseases. Such research centers on discovering or understanding existing pathogens in animals that have the potential to cause diseases in humans. Much of this research involves not only humans but also the animals that humans co-exist with and rely on. Findings may be framed as estimates of the risk of viral contagion from animals to humans (spillover) and potential outbreaks. Such findings can be particularly complex to communicate because of the social, economic, and political implications and perceptions of the risk of outbreaks.

This commentary discusses these complexities and challenges in disseminating findings on viral hemorrhagic fevers in bats in Sierra Leone. The findings were the result of research conducted under the Ebola Host Project (EHP) in Guinea, Sierra Leone, and Liberia. All 3 countries experienced devastating Ebola outbreaks, and the discovery of a new ebolavirus was part of efforts to build systems for prevention, detection, and response to future outbreaks under the Global Health Security Agenda. However, the process of disseminating research findings on discoveries of a new species of ebolavirus presented unique social and political challenges in managing the information and how it would be released to the affected communities, nationally and globally. This commentary describes these experiences and lessons learned and proposes elements that could inform a framework to guide processes of publicizing sensitive research findings on new zoonotic viruses.

Dissemination of findings on a new ebolavirus presented social and political challenges in managing the information and its release to affected communities.

## THE EBOLA HOST PROJECT

In response to the 2014–2016 Ebola virus outbreak, in 2015, the United States Agency for International Development (USAID) supported the EHP as part of an effort to strengthen zoonotic disease surveillance under its Global Health Security Agenda programs in 3 Ebola-affected countries. EHP, which was part of the USAID-supported PREDICT Project, was a research project that aimed to identify the range of possible animal hosts for all filoviruses as well as the human behaviors and conditions associated with the increased likelihood of spillover into humans and other animals.^4^ Such information is believed to be a key aspect of reducing the risk of future outbreaks. The project also aimed to increase national capacities for a multisectoral One Health approach for zoonotic disease surveillance, outbreak preparedness, laboratory systems strengthening, and workforce development.

The EHP was implemented similarly in all 3 countries. Strong local engagement was fostered through partnerships with local government and academic research institutions and training teams, often young graduates of science programs from those institutions, to collect data. The project also took a similar approach to community engagement in all 3 countries. This engagement took the form of smaller meetings with local leaders to create awareness and buy-in for the project, identification of community members who would work with the project during data collection, and larger meetings to inform community members about what the project would be doing and how.

After obtaining all the necessary permissions and commitments to collaborate, the project would plan and implement data collection and capture and sample animals. In each community, several types of wildlife (bats, rodents) were humanely captured. Samples collected from each animal included blood, oral and anal swabs, and urine if possible. The animal was then released. Samples were collected in duplicate. In Sierra Leone, over 10,000 animals were safely sampled and tested. One set of samples was stored at a designated laboratory in-country, and the other set was shipped to University of California (UC) Davis for further testing.

The project also included methods aimed at better understanding the human behavioral characteristics that increased the risk of exposure to hemorrhagic viruses. Group discussions, interviews, and observations were conducted with communities adjacent to sampling sites for this purpose.

**FIGURE 1:**
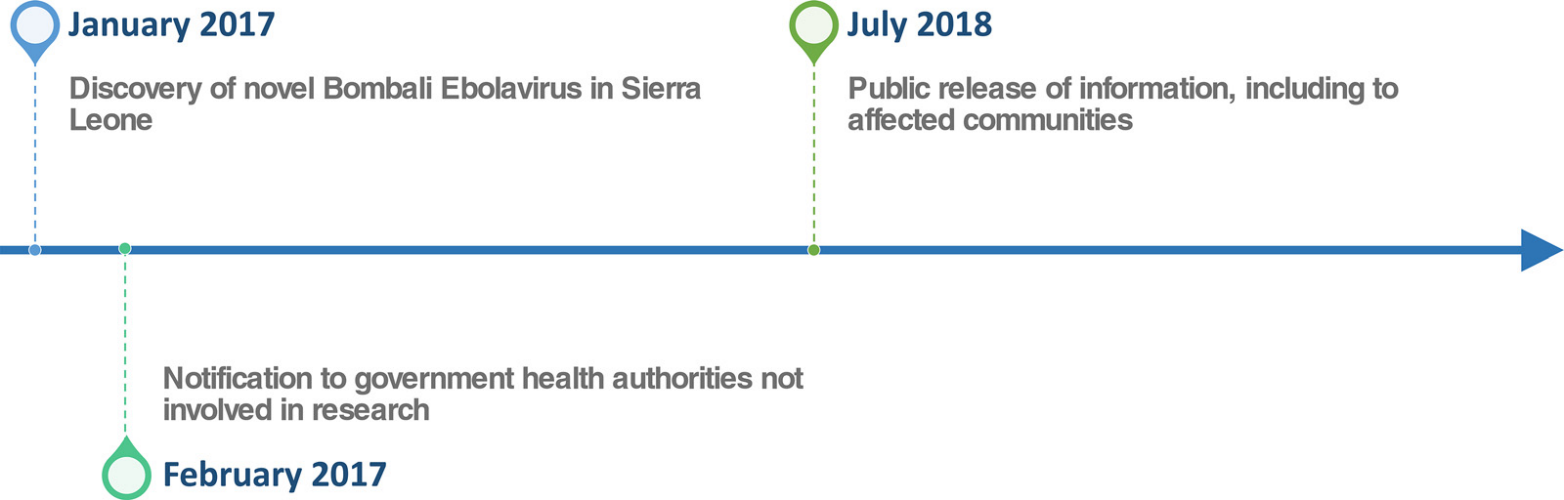
Timeline of Findings on New Ebolavirus in Sierra Leone

The achievements of the EHP were captured in news and scientific articles documenting findings of a new strain of the ebolavirus (Bombali virus),^5^ Marburg virus^6^ in bats in Sierra Leone, and the discovery of Ebola Zaire virus in a bat in Liberia.^7^ The scientific significance of these achievements was 2-fold. The first was the discovery of an entirely new species of ebolavirus (*Bombali ebolavirus*) in bats before any known human or animal illness or deaths, and the second was the discovery of a known and deadly human pathogen (Marburg virus/MARV) in bats for the first time in West Africa in Sierra Leone, more than 2,500 km from any known endemic area. However, before these findings became global news, host governments, donors, and researchers needed to consider how and when this information should be shared with the communities where samples were collected and the wider public. In the case of the *Bombali ebolavirus* discovery in Sierra Leone, these considerations led to a significant amount of time between the virus’ discovery and the point at which information about the discovery was publicized (Figure 1).

## SOCIAL AND POLITICAL CONSIDERATIONS AROUND DISSEMINATING RESEARCH FINDINGS

The amount of time that passed between the discovery of the Bombali virus and the publicization of information reflects the time needed to navigate social and political questions and reach consensus on a contextually appropriate process for dissemination. The EHP prioritized government leadership in the dissemination of results. In practice, this meant that laboratory detection and confirmation of a viral finding was followed by meetings with national government officials to discuss the findings and their implications. Decisions on whether, when, and how findings would be released were ultimately those of the national government.

However, news of the *Bombali ebolavirus* finding initially raised significant questions around the potential social implications and consequences of the discovery. Specifically, we were concerned about the potential of findings to amplify collective memories of devastation and loss around the relatively recent outbreak and undermine the progress that communities had demonstrated responding to the outbreak and ultimately moving forward. Furthermore, as we began working with the various levels of government to consider a dissemination strategy, the political implications of the finding were illuminated. The various issues can be broadly categorized into the following 3 domains.

News of the *Bombali ebolavirus* finding raised significant questions around the potential social implications and consequences of the discovery.

### Questions of Social Consequences and Behavioral Responses

Our first consideration around the *Bombali ebolavirus* finding was how the finding would affect communities. The virus was found in bats that resided in the roofing structures of homes. These homes were also in communities that experienced some of the highest incidences during Sierra Leone’s ebolavirus outbreak. These factors raised the possibility of fear, panic, and re-traumatization.

The provision of information in a clear and empowering manner was identified as one means of mitigating potentially negative emotional and psychological impacts. However, there were few examples of how to go about this and even fewer communication materials that articulated concepts such as the potential for viruses to pass from animals to humans or provided contextually appropriate messages and imagery.

A second issue we grappled with was that of social and contextually appropriate actions to recommend. It was widely recognized that the logical response to learning of this vulnerability would be for people to attempt to remove bats in their houses either by renovating their houses or by catching or killing bats. These response options pointed to underlying political and economic drivers of how and where people lived that are known to increase vulnerability to other zoonotic diseases such as Lassa fever in Sierra Leone.^8^ However, neither the government, the community, nor the EHP was equipped to provide structural solutions such as building better quality housing or moving people from the area to reduce exposure to bats. Moreover, other rational responses such as trying to kill bats could put people at greater risk of exposure to this virus while creating unintended ecological consequences. Significant consideration was given to contextually appropriate behavioral responses that could be safely recommended in the absence of options for addressing the structural inequities that continue to expose communities to risk.

### Questions of Intersections With National Politics

Sierra Leone was declared free of Ebola on November 7, 2016. The *Bombali ebolavirus* finding occurred less than a year after the official end of the outbreak. The politicization of the early stages of the outbreak and initial responses to the outbreak by the country’s 2 main political parties has been documented.^9^ They included rumors that an initially slow and inadequate response was motivated by the outbreak’s origins in a part of the country controlled by the opposition party. The government’s ability to make headway in the response, end the outbreak, and steer the country through recovery was an important overarching narrative in the country’s experience. The then President of Sierra Leone instituted a recovery plan targeting all sectors of society. The culmination of this recovery plan also coincided with campaigns for a national election. The narrative of overcoming the outbreak, recovery, and moving on stronger appeared to be complicated by the narrative of a scientific discovery of a new species of ebolavirus. The resolution of these conflicting narratives took a lot of time and consideration.

The findings ultimately were released after the election and under different government leadership. A reconciliatory narrative between government leadership and scientific discovery guided the process: The country’s disease systems had progressed to enable early detection of this virus in bats, long before it might be harmful to humans.

### Questions of Global Scientific Norms and Practice

In addition to country-level social and political implications, questions arose about global-level scientific research practice. One such question raised ethical considerations around sharing results indicating potential risk in the absence of further information to qualify that risk. Even when a pathogen is known to cause human disease, identification in a host reservoir does not mean that it is a public health threat. Therefore, providing such information when the immediate public health implications are not clear can raise more questions than are resolved.

Questions arose about global-level scientific research practice, including ethical considerations around sharing results about potential risk without information to qualify that risk.

Other questions touched on scientific practices, such as naming the virus after the town in which it was discovered. The global public health sector moved away from such practice following the H1N1 pandemic, but research scientists continue to take different approaches.^10^^,^^11^ Although government leaders ultimately determined it was not only socially acceptable but desirable to name the new virus after the town in which it was discovered, there is still a need for further consideration and evaluation of impact.

These social and political issues were multilevel and multidisciplinary. Context-specific questions of practice became intertwined with those of global best practice. There were no easy answers nor a clear way on how to proceed. Each issue required extensive consideration and negotiation by the Government of Sierra Leone, in-country project managers, and the U.S. Mission in Sierra Leone to navigate. This process took a significant amount of time and contributed to the delay in publicizing information.

## PROCESS OF DISSEMINATING RESEARCH RESULTS

Overcoming political issues associated with research findings translated into government leadership of the dissemination process. The approval to release information around the discovery of *Bombali ebolavirus* ultimately came from the office of the President of Sierra Leone. Research findings were presented at a high-level meeting with senior government officials from various ministries and led by the president. This approval catalyzed what were previously nascent plans for a multiphased public dissemination process. The dissemination plan had 3 components: a central meeting of district health personnel and media, a meeting of local leaders and residents in the community where the virus was detected, and global dissemination of findings beyond the country level.

**FIGURE 2:**
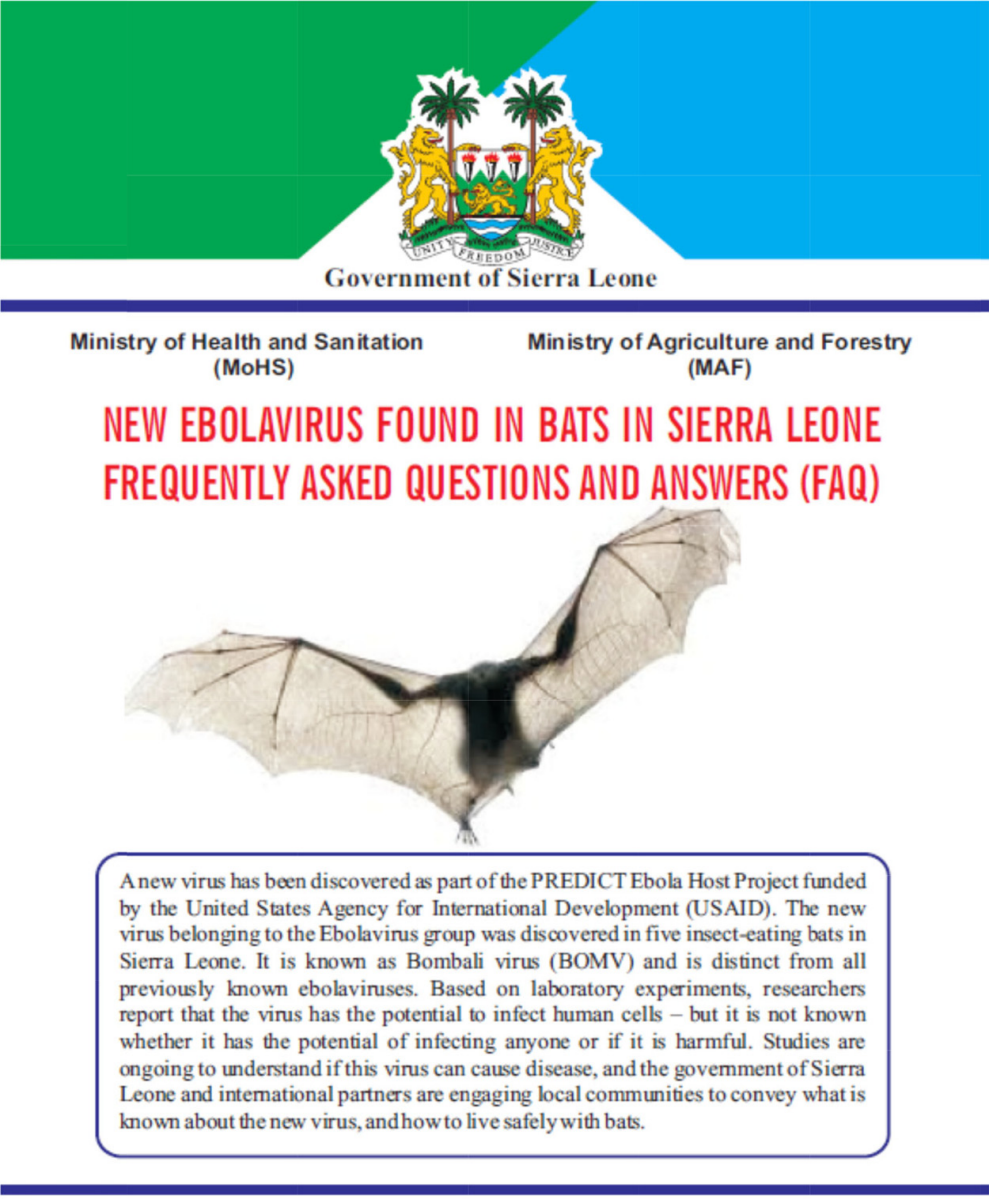
Cover of Government of Sierra Leone's Information Pamphlet on New Ebolavirus That Was Distributed to the Public

With political will behind the dissemination of results, we devised a communication strategy to address some of the social and behavioral issues identified. Communication aids such as talking points and draft press releases were developed in collaboration with the Ministry of Health and Sanitation and the Ministry of Agriculture and Forestry for dissemination to the public (Figure 2). However, it was agreed that more specific communication tools akin to those used in health promotion were needed for the meetings with local leaders and residents.

**FIGURE 3:**
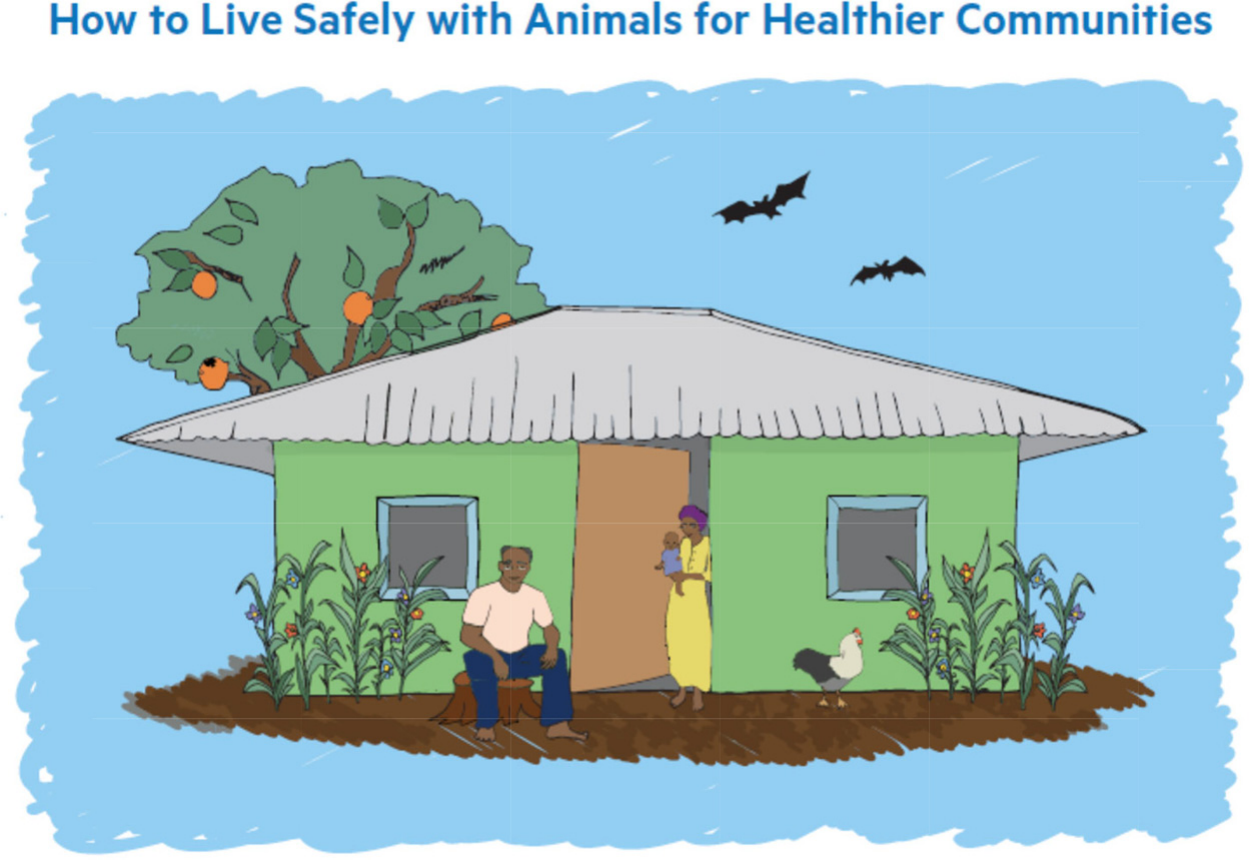
Cover of Living Safely With Animals, a Book Used by Local Health Officials in Sierra Leone to Engage the Community in Discussions on Research Findings on New Ebolavirus

For this reason, we engaged another USAID-funded program, Breakthrough ACTION, which focused on social and behavioral communication around the zoonotic disease. Breakthrough ACTION worked with the EHP to develop early drafts of a picture book on communication with bats. Bringing a social and behavioral communication partner into this process allowed us to apply an evidence-based approach to clarifying key messages, adapting imagery, and piloting and revising the book into something that could be used by local health officials to engage communities in discussions around the findings. A key adaptation resulting from these processes was the need to broaden the focus of the book beyond bats to one that promoted safe behaviors with all animals routinely encountered in the community. The emphasis became “Living Safely with Animals” (Figure 3). This shift in emphasis was agreed on as being necessary to reduce the likelihood of behaviors, such as killing bats, that could increase the risk of exposure to the virus. The book also provided something tangible to be left with health promotion and social mobilization teams to use in the ongoing work.

Bringing a social and behavioral communication partner into the information dissemination process allowed use of an evidence-based approach to clarify key messages to the public.

## INSIGHTS FROM OUR EXPERIENCES WITH DISSEMINATION

Our experience of navigating the social and political challenges around the discovery of *Bombali ebolavirus* in Sierra Leone can inform future research on emerging zoonotic diseases as part of the Global Health Security Agenda. This experience pointed to the tensions that can arise between discoveries of emerging viruses with zoonotic potential and their impact on the people in direct proximity to the ecosystems in which they are found, as well as the governments charged with protecting the health of those people. This experience also demonstrated that the interests of the global scientific community must be balanced, negotiated, and navigated alongside those of national governments and the communities that bear the burden of the consequences of this information. Similarly, national governments must also be open to receiving scientific information with potential health impacts and formulating timely, contextually appropriate responses. This is not only important for navigating the process of publicizing findings in ways that mitigate fear but also empowers people and societies to adopt appropriate behaviors to protect their health. Future research may benefit from consideration of the following steps.

### Prioritize Dissemination of Findings as a Distinct Step Within a Project With a Detailed Plan

The complicated set of social and political factors previously described in combination with the various activities and resources that were required to prepare for dissemination represented a substantial effort. Much of this occurred ad hoc because there was no written plan or protocol to guide this stage of the research project. A dissemination plan with an associated budget can be a helpful tool from the onset of the project both as a basis of coordination among stakeholders and as a basis for ongoing adaptation to social and political contexts. Wherever possible, the research team should consider incorporating the dissemination plan into the research protocol from the beginning of the project with a view toward adapting as needed and working alongside government counterparts as the project progresses.

A dissemination plan can be a helpful tool from the onset for coordinating stakeholders and for ongoing adaptation to social and political contexts.

### Prioritize Understanding and Supporting the Role of Various Government Leaders in the Dissemination Process

Prioritizing government leadership means understanding what is meant by “government.” There may be a risk for those without a country-level understanding to use the term “government” monolithically and not recognize the multiple levels of government from community to national level and the embedded hierarchies for decision making. This is especially important when research projects say “government is involved.” The government officials involved in the research may not be the same officials who have the authority to release research findings. This may require continued engagement of higher-level authorities or the departments involved in research. Having a nuanced view of a country’s government structure is also important for thinking more broadly about the range of potential support needed for their leadership in decision making. This means thinking beyond national-level press releases to consider, for example, what a district health manager needs to communicate to local communities.

### Prioritizing the Perspectives and Potential Reactions of Communities When Framing Information and Recommendations

Prioritizing potential social impacts and behavioral responses to research results was key to determining not only what people were told about the finding but what they could do to protect themselves from perceived risks. Prioritizing community perspectives requires careful consideration of a range of responses and the potential for misunderstanding. Ultimately, a comprehensive community engagement strategy is crucial for the dissemination of research results while countering potential misconceptions, misinformation, myths, and fake news. However, completely preventing misinformation may be impossible. For this reason, social listening, rumor monitoring, and plans for response should be part of a community engagement strategy. This strategy includes considering scenarios whereby misunderstandings could result in various forms of backlash. In this instance, the safety of community members who have been involved in the research also needs to be considered.

The Table lists activities that can be considered as part of a comprehensive framework for disseminating findings on emerging infectious diseases in contexts with histories of outbreaks.

**TABLE:** Illustrative Activities for Planning Dissemination

**Phase of Dissemination**	**Potential Activities at Community, National, and Global Levels**
Pre-dissemination	Create dissemination plans with government stakeholders at various levels – (i.e., community, district, regional and national)
	Consider behaviors and actions that people can reasonably take based on findings
	Prepare locally appropriate information materials in collaboration with Ministries of Health, Agriculture/Veterinary Services, and Health Education/Social Mobilization
	Prepare rumor monitoring and response plans at various levels
	Consider safety and security plans for research staff and community members
	Consider scenarios for sensitive findings
Dissemination and Post-dissemination	Review conduct and dissemination of research with government counterparts
	Implement and monitor rumor management system
	Assure ongoing communication with government counterparts at various levels

## CONCLUSION

Discovering emerging zoonotic pathogens before they pose threats to humans is an essential aspect of detecting and preventing future outbreaks and pandemics. The ongoing COVID-19 pandemic has highlighted the importance of these efforts and the need for ongoing research on emerging pathogens. However, the current COVID-19 pandemic is also showing us that pandemics and research on emerging zoonotic viruses have social and political implications.

Our experience in Sierra Leone highlighted some of these implications, and it suggests that greater consideration should be given to the overall approach and processes involved in the dissemination of research findings. Dissemination should consider the social and political contexts of stakeholders, such as national and local governments, local communities, and global scientific research communities. While these groups may overlap in terms of an overall goal of protecting public health, they have distinct responsibilities and accountabilities.

In Sierra Leone, these distinctions emerged more so in the dissemination phase and presented challenges to publicizing information. The process of dissemination should prioritize local community engagement and multilevel communication strategy. This prioritization may require collaboration with entities with a specialized skill set in social and behavioral communication to develop or adapt communication tools to incorporate new research findings and implications for health behaviors. Communication preparedness plans with risk mitigation elements, including engagement with various levels of government and community members, should be built into projects, along with corresponding budgets.

The interdisciplinary nature of these considerations requires an operational framework for research dissemination that involves those with social and behavioral expertise working alongside virologists, government leaders, and communities. Such a framework must also capture best practices and inform wider efforts related to community-based research for health security. If effectively implemented, dissemination of research findings on emerging zoonotic viruses can be government owned and led while applying practices that mitigate fear and build community trust for prevention, detection, and response to future outbreaks and pandemics.

## Notes

**Peer Reviewed**

**First published online:** July 15, 2021.
